# Mineral and Phenolic Composition of *Erodium guttatum* Extracts and Investigation of Their Antioxidant Properties in Diabetic Mice

**DOI:** 10.1155/2022/4229981

**Published:** 2022-09-19

**Authors:** Kaoutar Benrahou, Hanae Naceiri Mrabti, Saad Fettach, Mohamed Reda Kachmar, Mostafa Kouach, Jean-François Goossens, Latifa Doudach, Shafi Mahmud, Mohammed Merae Alshahrani, Ahmed Abdullah Al Awadh, Abdelhakim Bouyahya, My El Abbes Faouzi

**Affiliations:** ^1^Laboratory of Pharmacology and Toxicology, Bio Pharmaceutical and Toxicological Analysis Research Team, Faculty of Medicine and Pharmacy, Mohammed V University in Rabat, BP 6203, Rabat, Morocco; ^2^Faculty of Sciences, Health and Environment Laboratory, Plant Protection Team, Moulay Ismail University, BP 11201, Zitoune, Meknes, Morocco; ^3^Univ. Lille, CHU Lille, ULR 7365-GRITA-Groupe de Recherche sur les formes Injectables et les Technologies Associées, F-59000 Lille, France; ^4^Department of Biomedical Engineering Medical Physiology, Higher School of Technical Education of Rabat, Mohammed V University in Rabat, BP 6203, Rabat, Morocco; ^5^Division of Genome Sciences and Cancer, The John Curtin School of Medical Research and The Shine-Dalgarno Centre for RNA Innovation, The Australian National University, Canberra, ACT 2601, Australia; ^6^Department of Clinical Laboratory Sciences, Faculty of Applied Medical Sciences, Najran University, 1988, Najran 61441, Saudi Arabia; ^7^Laboratory of Human Pathologies Biology, Faculty of Sciences, Mohammed V University in Rabat, Morocco

## Abstract

*Erodium guttatum* is widely used in folk medicine in many countries to treat various ailments such as urinary inflammation, diabetes, constipation, and eczema. The aim of this study is the determination of mineral and phenolic compounds of *E. guttatum* extracts as well as the investigation of their antidiabetic and antioxidant properties. The mineral composition was determined by the methods of inductively coupled plasma atomic emission spectroscopy analysis. Phytochemical contents of total polyphenols, total flavonoids, and catechic tannins were estimated by colorimetric dosages. The phenolic composition was identified by high-resolution mass spectrometry (HRMS) analysis. The antioxidant activity of *E. guttatum* extracts was measured *in vitro* by five methods (DPPH, ABTS, FRAP, H_2_O_2_, and xanthine oxidase) and *in vivo* by assaying the malondialdehyde marker (MDA), superoxide dismutase (SOD), catalase (CAT), and glutathione (GSH). The obtained results showed that the root plant material is rich in minerals such as k, Ca, and Mg. The methanolic extract of *E. guttatum* is the richest in polyphenols (389.20 ± 1.55 mg EAG/gE), tannins (289.70 ± 3.57 mg EC/gE), and flavonoids (432.5 ± 3.21 mg ER/gE). Concerning the ESI-HRMS analysis, it showed the presence of numerous bioactive compounds, including shikimic acid, rottlerine, gallic acid, and vanillic acid. Moreover, the aqueous and alcoholic extracts of *E. guttatum* exhibited antiradical and antioxidant activity in five tests used, with the best effect of the methanolic extract. Moreover, findings showed that *in vivo* investigations confirmed those obtained *in vitro.* On the other hand, *E. guttatum* showed important antidiabetic effects *in vivo*. Indeed, diabetic mice treated with extracts of *E. guttatum* were able to significantly reduce MDA levels and increase the secretion of enzymatic and nonenzymatic antioxidants (SOD, CAT, and GSH, respectively). However, the antioxidant activity of the extracts might be attributed to the abundance of bioactive molecules; as results, this work serves as a foundation for additional pharmacological research.

## 1. Introduction

Oxidative stress is a consequence results from the oxidative balance deregulation between the production and the release of free radicals, which can cause certain damage at the tissue level by the oxidation of bimolecular such as enzymes, proteins, DNA, and lipids [1]. Excessive production of reactive oxygen species (ROS) is considered as a major risk factor of several diseases such as aging, atherosclerosis, diabetes, rheumatoid arthritis, carcinogenesis, and neurodegenerative diseases [2]. Interestingly, cells can produce many antioxidant defense enzymes to trap and neutralize ROS, including catalase, superoxide dismutase, glutathione reductase, glutathione peroxidase, and some antioxidant molecules (uric acid, glutathione, albumin, protein-SH groups, bilirubin), as well as some vitamins such as ascorbic acid, *α*-tocopherol, and carotenoids [3].

Most organisms have evolved antioxidant defense and repair systems to protect them against oxidative damage. However, these systems are insufficient to completely prevent the damage [4]. Therefore, it is necessary to develop natural nontoxic antioxidants to protect the human body from free radicals and delay the progression of many chronic diseases [5–7]. For a long time, medicinal plants have been proven to be very rich in antioxidant molecules other than vitamin C, vitamin E, and carotenoids. The antioxidant effect is due to their richness in phenolic components, such as flavonoids, phenolic acids, and phenolic diterpenes [7].

Morocco is endowed with a great climatic and biological variety in which plants can be cultivated or grown spontaneously. Morocco is very popular due to its large and very diverse flora; out of 4200 spontaneous species, about 1500 introduced species have been recorded, some of them listed in Moroccan pharmacopoeia [8]. Plants always had a crucial role, especially for their important therapeutic role in treatment. Modern medicine has made great strides throughout the last century. Traditional medicine is still important in the lives of many people around the world. Indeed, the pharmacopoeia contains approximately 25,000 herbs, and approximately 50% of all pharmaceutical products on the market are of natural origin [9].

Numerous studies show that medicinal plants are frequently used, including those from the Geraniaceae family. Ethnopharmacological data indicated that several species of *Erodium* are traditionally used in Turkey to treat various diseases such as urinary inflammation, diabetes, constipation, and eczema [10]. In other countries, it is used as an astringent and antiseptic tea [11]. The genus *Erodium* exists on all continents, and it is especially very widespread in the Mediterranean region, and more than 60 species can be found. *Erodium* contains annual or perennial herbaceous beds and it is characterized by five fertile stamens and five staminodes. The leaves are pinnate or undivided into pinnatifid to pinnatisect. Leaf size and shape can vary between populations, and variation within populations is generally evident [12].

Among the species of the genus *Erodium*, we found *Erodium guttatum*, which is used in traditional medicine, while it is less explored for its biological and pharmacological properties. Indeed, antioxidant studies have not been performed on *E. guttatum*. In addition, *in vitro* antioxidant and antimicrobial activities are the only studies performed on *E. guttatum* [13]. Additionally, this work is aimed at determining the mineral and phenolic compounds of *E. guttatum* root extracts as well as the investigation of their *in vitro* and *in vivo* antioxidant and antidiabetic effects.

## 2. Materials and Methods

### 2.1. Plant Material and Preparation of Extracts

The fresh roots of *E. guttatum* were harvested in February 2018 in the northeast regions of Oujda, Morocco. After being identified by botanist Professor Mohammed Fennan, the specimen was deposited at the Herbarium of Botany of the Scientific Institute of Rabat under the reference number 110970. The roots of *E. guttatum* were dried in the shade at room temperature and then ground using an electric grinder. The ground material obtained was stored in a dry place and protected from moisture and light until its use.

To prepare the ethanolic and methanolic extracts, 30 g of plant material (powder) was macerated successively with 300 mL of ethanol and methanol for 48 h with mechanical stirring. The aqueous extract was infused with boiling water for 1 hour and allowed to cool. The extracts were then filtered, and the filtrate obtained was evaporated using a rotary evaporator and then lyophilized.

### 2.2. Mineral Composition

The mineral composition (Ca, Mg, Mn, Fe, Zn, K, Na, and P) was determined using an inductively coupled plasma atomic emission spectroscopy (ICP AES, JobinYvonUltima 2). The 150 mg of the plant and leaf powders were washed with a 2 mL of HNO_3_ acid (70%) mixture in a Teflon beaker, before being incinerated at 110°C. Then, 0.5 mL of hydrofluoric acid (HF) was added, and the covered beaker was placed on a sand bath. The sample mixture was heated until a clear solution was obtained. After removing the cover, the mixture evaporated to dryness. Finally, 2 mL of HCl acid was added and the residue was extracted with 25 mL of 2.0 M HCl.

### 2.3. Determination of Total Phenols

Quantification of the total polyphenol is performed using the Folin Ciocalteu (phosphotungstic and phosphomolybdic) reagent described in the literature [14]. Indeed, 0.25 mL of each concentration extract (1 mg/mL) was mixed with 2.5 mL of Folin Ciocalteu reagent (10 times diluted in distilled water). The reaction mixture was stirred vigorously, and then, 4 mL of 7.5% (*W*/*V*) Na_2_CO_3_ sodium carbonate was added. The mixture was incubated in a water bath at 45°C for 30 minutes, and the absorbance was recorded by a spectrophotometer at 765 nm. Under the same operating conditions, the standard range of gallic acid was carried out at different concentrations (1.95, 3.9, 7.81, 15.625, and 31.25 *μ*g/mL), and the results were expressed in milligrams of gallic acid equivalent in grams of extract (mg EAG/1 g extract).

### 2.4. Total Flavonoid Determination

The flavonoid content was estimated using the colorimetric method with aluminum trichloride (AlCl_3_) and sodium hydroxide (NaOH) as described by Dewanto et al. [15]. In brief, 0.5 mL of each 1 mg/mL concentration sample was mixed with 3.2 mL of distilled water and 0.15 mL of 5% (*w*/*v*) NaNO_2_ sodium nitrite solution. The reaction mixture was incubated for 5 minutes. 0.15 mL of aluminum trichloride (AlCl_3_) was added, followed by a second additional incubation for 6 minutes. Finally, 1 mL of sodium hydroxide NaOH (1 M) was added, and the mixture was incubated at room temperature for 30 minutes while shaded. The absorbance was measured at a wavelength of 510 nm. As a standard range, rutin was used at different final concentrations (50, 100, 150, 200, 250, 300, 350, and 400 *μ*g/mL), and results were expressed in milligrams of gallic acid equivalent per gram of extract (mg ER/1 g Ex).

### 2.5. Dosage of Condensed Tannins

The tannin content was estimated by the method of vanillin as described by Julkunen-Tiitto [16]. However, 50 *μ*L of each 1 mg/mL concentration extract was mixed with 1.5 mL of 4% vanillin (prepared with methanol), and then, 750 *μ*L of concentrated HCl hydrochloric acid was added. The reaction medium is stirred vigorously and incubated at room temperature and in the dark for 20 minutes. The absorbance was read at a wavelength of 500 nm, and the calibration curve was established with catechin at concentrations of (50, 100, 200, 300, 400, 500, 600, 800, and 1000 *μ*g/mL) under the same conditions.

### 2.6. Determination of Chemical Composition by HRMS Analysis

HRMS analysis was performed by ionization (ESI) mass spectrometry (MS). The extracts were dissolved to a final concentration of 1-2 pmol/*μ*L in methanol. Compounds were measured in negative and positive modes by full mass scanning (*m*/*z* 50 to 1000) using a Thermo Scientific Orbitrap Exactive mass spectrometer equipped with a heated electrospray ionization source (HESI-II). The instrument parameter settings are as follows: sheath gas 10 in positive mode and 20 in negative mode (arbitrary units), spray voltage 3.5 kV and 3 kV in positive and negative mode, respectively, with a 275°C capillary temperature. Mass spectra were collected at a resolution of 100,000. Data processing was performed using the associated software, Xcalibur 2.2 and Exactive 1.1.

### 2.7. Antioxidant Activity

#### 2.7.1. Radical Scavenging Assay with DPPH

DPPH (2,2-diphenyl-1-pierylhydrazyl) is a synthetic free radical of intense violet color. In the presence of free radical scavengers, purple DPPH is reduced to yellow DPPHH (2,2-diphenyl-1-picryl-hydrazine) [17]. In brief, 1.25 mL of each extract was mixed with 0.25 mL of methanol prepared DPPH solution and incubated in the dark at room temperature for 30 minutes, and the absorbance was recorded at 517 nm against a control sample. The percentage of antiradical activity is calculated according to the following formula:

(1) $$\begin{array}{c} I\%=\left[\frac{\left(\text{Absorbance Negative Control}-\text{Absorbance Sample}\right)}{\text{Absorbance Negative Control}}\right]*100. \end{array}$$

#### 2.7.2. Ferric Reducing Power Assay (FRAP)

FRAP is an antioxidant test that measures the power of reducing ferric iron to ferrous iron [18]. However, the measure of the reducing power was determined by mixing 0.5 mL of each extract at a concentration of 1 mg/mL with 1.25 mL of phosphate buffer solution (0.2 M, pH 6.6) and 1.25 mL of aqueous solution of potassium ferricyanide [K_3_Fe (CN) _6_] at 1%. The mixture was incubated in a water bath at 50°C for 20 minutes, then 1.25 mL of trichloroacetic acid (10%) was added to stop the reaction. The tubes are centrifuged at 3000 rpm for 10 minutes. An aliquot (1.25 mL) of supernatant is combined with 1.25 mL of distilled water and 0.25 mL of a 0.1% aqueous FeCl_3_ (ferric chloride) solution. Ascorbic acid is used as a standard, and the final results are expressed in milligram equivalents of ascorbic acid per gram of extract.

#### 2.7.3. Trolox Equivalent Antioxidant Capacity (TEAC) Assay

For the ABTS test (2,2′-Azino-bis (3-ethylbenzothiazoline-6-sulfonic acid)), the cationic radical ABTS was generated by mixing 10 mL of the solution of ABTS (2 mM) in H_2_O and 100 *μ*L of the potassium persulfate solution (70 mM). The mixture is preserved for 16 hours. The resulting solution was diluted with methanol to obtain an absorbance of 0.70 to 734 nm. From this solution, 2 mL of diluted ABTS were mixed with 200 *μ*L of each extract at a concentration of 1 mg/mL and allowed to react for 1 minute. The absorbance was measured at 734 nm. Trolox is used as a standard, and the final results are expressed in milligram equivalents of Trolox per gram of extract [19].

#### 2.7.4. H_2_O_2_ Test

The activity of scavenging H_2_O_2_ was determined by the Muruhan et al. [20] method. A solution of H_2_O_2_ (40 mmol/L) was prepared in phosphate buffer (pH 7.4). The concentration of H_2_O_2_ was determined spectrophotometrically from the absorption at 230 nm. Indeed, 1 mL of the extract or of the standard (ascorbic acid) at different concentrations was added to the H_2_0_2_ solution (0.6 mL, 40 mmol/L). After 10 min of incubation, the absorbance of H_2_O_2_ was determined at 230 nm against a blank solution containing a phosphate buffer without H_2_O_2_. The scavenging percentage of H_2_O_2_ for the extract and ascorbic acid was calculated using the following equation:

(2) $$\begin{array}{c} \%=\left[\frac{\left(A_{0}–A_{1}\right)}{A_{0}}\right]\times 100, \end{array}$$

where *A*_0_ was the absorbance of the control and *A*_1_ was the absorbance in the presence of the sample or standard.

#### 2.7.5. Xanthine Inhibition Assay

Inhibition of xanthine oxidase (xo) was determined by Umamaheswari et al. [21]. However, 1 mL of the extract or standard (allopurinol) was mixed with 1.9 mL of phosphate buffer (pH 7.5), 0.1 mL of enzyme solution (0.2 units/mL), and 1.0 mL of 0.5 mM xanthine solution. The reaction mixture was incubated for 15 minutes at 25°C. Subsequently, the enzymatic reaction was stopped with 1 mL of 1 M HCl, and the absorbance of the reaction mixture was measured at 295 nm against a control solution prepared in the same manner without adding any enzyme solution. The percentage of XO inhibition was calculated as follows:

(3) $$\begin{array}{c} I\left(\%\right)=\left[\left(\frac{\left(\text{Ac}-\text{Acb}\right)-\left(\text{As}-\text{Asb}\right)}{\left(\text{Ac}-\text{Acb}\right)}\right)\times 100\right], \end{array}$$

where Ac denoted the absorbance of the control, Acb the absorbance of the control blank, As the absorbance of the sample, and Asb the absorbance of the sample blank.

#### 2.7.6. Animals

Healthy male mice (20–25 g) were used in the experiments. Animals were kept in cages at the Rabat Faculty of Medicine and Pharmacy (Morocco) and maintained in a controlled room at 22 ± 02°C with a 12 h light–dark cycle with free access to water and standard food.

#### 2.7.7. Ethics Approval

The study was conducted in accordance with the principles described in the “Guide for the Care and Use of Laboratory Animals”, 8th edition, prepared by the National Academy of Sciences (National Research Council of the National Academies, 2011). Every effort was made to minimize animal suffering and the number of animals used. Mohammed V University in Rabat granted ethics approval.

#### 2.7.8. Experimental Design

*(1) (1) Development of Type 2 Diabetes*. The antihyperglycemic effect of *E. guttatum* extract was studied in hyperglycemic mice induced by HFD. Indeed, after 15 days of accommodation, excluding normal control with a normal diet, the other mice were fed a high-fat diet. The diet was prepared daily as described in the literature [22]. It consists of standard diet carbohydrates at 50%, protein at 18%, fat at 30%, and salt and vitamins at 2%. After 4 weeks, the mice received an intraperitoneal injection of 100 mg/kg of streptozotocin dissolved in a citrate buffer (0.1 M, pH 4.5). Five days later, blood samples were taken from the tail vein of the fasting mice after the induction of STZ. Mice with a fasting blood sugar level greater than 126 mg/dL were considered T2DM and included in the study.

*(2) (2) Experimental Design*. After induction of diabetes, the thirty diabetic mice were divided into five groups of six mice each. The untreated diabetic group (negative control); the diabetic group treated with metformin at a dose of 300 mg/kg (positive control); and the aqueous, ethanol, and methanol extracts were given to the other three diabetic groups at a dose of 200 mg/kg [23, 24]. The normal group containing the six nondiabetic mice was allowed free access to a normal diet and treated with saline.

#### 2.7.9. Preparation of Homogenates

At the end of the treatment, we extracted the organs (liver, kidney, and pancreas) for the determination of the markers of oxidative stress. Initially, we cut and ground the organs with 10% phosphate buffer (0.05 M, pH 7.4). Subsequently, the homogenate was centrifuged at 10,000 rpm for 10 min at 4°C. In the last step, we collected the supernatant and stored it as an aliquot at -20°C for analysis of oxidative stress parameters.

#### 2.7.10. Determination of Malondialdehyde (MDA)

The determination of malondialdehyde is based on the measurement in acidic and hot media of the MDA-TBA complex according to the Ohkawa et al.'s technique [25]. 100 *μ*L of the homogenate or MDA was mixed with 300 *μ*L of 0.6% thiobarbituric acid and 700 *μ*L of 1% phosphoric acid. The reaction mixture was then heated to 95°C for 30 minutes. After cooling, 2 mL of n-butanol was added and then centrifuged at 3000 rpm for 10 minutes. The absorbance of the MDA-TBA complex was determined spectrophotometrically at 535 nm.

#### 2.7.11. Superoxide Dismutase Assay

Superoxide dismutase (SOD) constitutes the enzymes of the first lines of defense against free radicals. They disproportionate the superoxide anion into hydrogen peroxide and oxygen. The assay is carried out according to the method of Beauchamp and Fridovich [26]. A reaction mixture was prepared by adding 50 mM phosphate buffer (pH 7.2), 0.25% triton x-100, 10 mM EDTA (pH 8), 120 mM L-methionine, 0.75 mM NBT, and at the end, 10 *μ*M of riboflavin. The reaction was performed at 25°C under a 15 W lamp for 10 min to induce the photoreaction of riboflavin and O_2_. The absorption is read at 560 nm, and the SOD (superoxide dismutase) activity is calculated according to the following equation:

(4) $$\begin{array}{c} \text{Inhibition}\%=\left(\frac{\text{Absorbance Blank}-\text{Absorbance sample}}{\text{Absorbance blank}}\right)\times 100. \end{array}$$

The SOD unit is the amount of enzyme that causes 50% inhibition of NBT reduction.

#### 2.7.12. Catalase Activity

Catalase activity was determined by the Aebi [27] method, measuring the disappearance of hydrogen peroxide (H_2_O_2_). The catalytic activity was expressed in *μ*mol of H_2_O_2_ per minute and per mg of protein. 780 *μ*L of phosphate buffer (100 mM, pH 7.5) was mixed with 20 *μ*L of homogenate. Then, the reaction was initiated by adding 200 *μ*L of hydrogen peroxide (H_2_O_2_) (500 mM). The decrease in optical density due to the decomposition of hydrogen peroxide after 1 min was measured against the blank without homogenate at 240 nm.

#### 2.7.13. Glutathione (GSH) Activity Assay

The glutathione level was measured according to the Maron method [28]. The assay is based on the reduction of 5.5-dithio-bis-2-nitrobenzoic acid (Ellman's reagent) by the (-SH) groups of glutathione. Indeed, the reaction mixture contained 200 *μ*L of TCA (5%) and 400 *μ*L of homogenate and was centrifuged at 12,000 g for 10 min, then 50 *μ*L of the supernatant was taken and added to 100 *μ*L of DTNB (6 mM) and 850 *μ*L of 50 mM phosphate buffer. The absorbance was read after 5 min at 412 nm.

## 3. Results

### 3.1. Mineral Composition

The mineral composition of *E. guttatum* roots is summarized in Table 1. Five macroelements (calcium (Ca^2+^), potassium (K^+^), magnesium (Mg), sodium (Na^+^), and phosphorus (P)) and fourteen microelements (boron (B), manganese (Mn), copper (Cu), iron (Fe^2+^), zinc (Zn), molybdenum (Mo), nickel (Ni), cobalt (Co), chromium (Cr), selenium (Se), lead (Pb), vanadium (V), arsenic (AS), and cadmium (Cd)) were analyzed. As can be seen, roots contain significantly more Ca^2+^ (28300.88 mg/kg), K^+^ (8861.61 mg/kg), Mg (2421.03 mg/kg), and P (1421.74 mg/kg), with minor content in Zn (334. 74 mg/kg), Na (228.26 mg/kg), Fe^2+^ (170.54 mg/kg), B (19.54 mg/kg), Mn (18.15 mg/kg), and Cu (15.64 mg/kg) and trace amounts of Se (0.01 mg/kg), Cd (0.12 mg/kg), and Mo (0.5 mg/kg).

### 3.2. Phenolic, Flavonoids, and Tannin Contents

The quantification of polyphenols, condensed tannins, and total flavonoids of *E. guttatum* extracts is presented in Table 2. In fact, the quantity of phenols is greater in the methanolic extract (389.20 ± 1.55 mg of GAE/g of extract), followed by the ethanol extract (348.1 ± 2.3 mg of GAE/g of extract), and aqueous (248.17 ± 1.81 mg of GAE/g of extract). The condensed tannins are also much more concentrated in the methanolic extract (289.70 ± 3.57 mg EC/g of extract) than in the ethanolic extract (243.3 ± 2.21 mg EC/g of extract) and aqueous (114.95 ± 2.60 mg EC/g of extract). The same observations were recorded for flavonoids, the methanolic extract (432.5 ± 3.21 of ER/g of extract), the ethanolic extract (417.5 ± 1.42 of ER/g of extract), and the aqueous extract (315.5 ± 4.50 of ER/g of extract).

### 3.3. Chemical Bioactive Compounds

**Figure 1:**
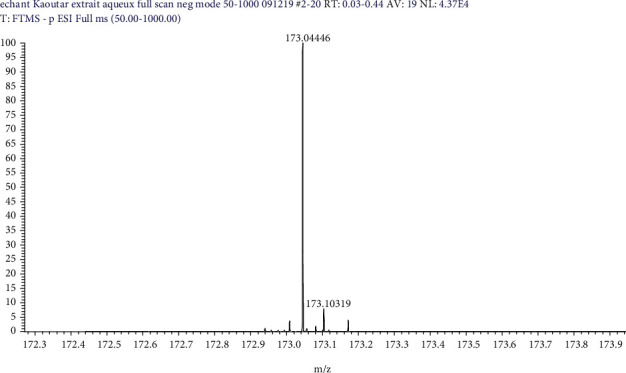
HRMS mass spectrum obtained in negative mode for shikimic acid in aqueous extract.

The compounds identified by the ESI-HRMS experiment are summarized in Table 3, the selectivity of HRMS (Figure 1) as well as molecular formulas, RDB (Rings and Double Bond Equivalents) unsaturation values, and mass accuracy. Direct analysis by electrospray ionization (ESI) in both negative and positive mode in full mass scanning (*m*/*z* 50 to 1000 amu) identified 10 compounds, summarized as follows: shikimic acid, rottlerin, rugulosin, and procyanidin C1 in EGA at *m*/*z* of (174, 516, 542, and 866). Sucrose, tiliroside, and vanillic acid have *m*/*z* values of 342, 594.13, and 168 in EGE, respectively; the presence of gallic acid at *m*/*z*; 170 and catechin (*m*/*z*; 290) in EGA, EGM, and EGE.

### 3.4. Antioxidant Activity

#### 3.4.1. *In Vitro* Studies

**Figure 2:**
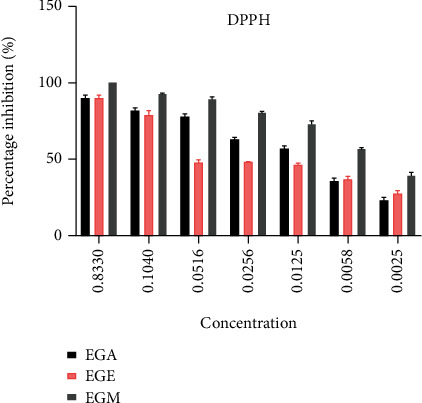
Percentage of DPPH radical inhibition by extracts of *E. guttatum*. EGA: *Erodium guttatum* aqueous extract; EGM: *Erodium guttatum* methanolic extract; EGE: *Erodium guttatum* ethanolic extract.

*(1) (1) Scavenging of the Free Radical DPPH*. The antiradical activity profile of each extract tested against the DPPH radical is presented in Figure 2. Indeed, these curves reveal that the antiradical power is proportional to the concentration of the extracts. We found that the percentage of inhibition increased as the concentration of the extracts increases.

From these data, the IC_50_ inhibitory concentration was determined by GraphPad prism 6 software, and it is shown in Table 4. The results obtained show that each extract tested has an interesting antifree radical power. Indeed, by comparing the IC_50_ values, it is observed that the methanolic extract has a significantly higher activity than aqueous and ethanolic extracts (*p* < 0.05). Thus, the ethanolic extract of *E. guttatum* has a significantly higher antiradical activity than the aqueous extract (*p* < 0.05).

*(2) (2) Iron-Reducing Power (FRAP)*. Ferric reducing power can serve as a significant indicator of antioxidant potential. The methanol extract showed better antioxidant activity. In addition, the methanolic extract has a reducing power of 540.2 ± 1.40 mg EAA/gE, which is significantly (*p* < 0.05) higher than that of the ethanolic extract (220.72 ± 1.05 mg EAA/gE). Thus, the aqueous extract showed significantly (*p* < 0.05) higher antioxidant activity than the ethanolic extract (Table 4). Furthermore, if we rank our extracts according to iron reduction potency with respect to ascorbic acid, we will obtain the following order: methanolic extract of *E. guttatum*, aqueous extract of *E. guttatum*, and ethanolic extract of *E. guttatum*.

*(3) (3) Antioxidant Power by ABTS*. The antioxidant activity of three tested extracts was evaluated using the ABTS method. The results of the extracts are presented in Table 4 and are expressed as TEAC (TROLOX Equivalent Antioxidant Capacity). Indeed, the results showed that methanolic extract has a significantly (*p* < 0.05) powerful activity of 228.68 ± 2.93 mg of Trolox ET/gE, while aqueous extract has an antioxidant power of 218.52 ± 1.34 mg of Trolox ET/gE, and ethanolic extract shows an activity of 189.13 ± 1.41 mg of Trolox ET/gE.

*(4) (4) Hydrogen Peroxide Trapping Test H_2_O_2_*. The scavenging effects of aqueous and alcoholic extracts of *E. guttatum* and ascorbic acid on the H_2_O_2_ radicals expressed by IC_50_ are shown in Table 4. The hydrogen peroxide H_2_O_2_ scavenging test showed that the aqueous extract exhibited the best antioxidant effect. By comparing the IC_50_ values, the aqueous extract (IC_50_ = 4.65 ± 0.7 *μ*g/mL) presented an activity that was significantly (*p* < 0.05) higher than that of ascorbic acid (IC_50_ = 5.98 ± 0.47 *μ*g/mL). Moreover, the ethanolic extract showed significantly (*p* < 0.05) higher activity than the methanolic extract.

*(5) (5) Xanthine Oxidase Test*. Xanthine oxidase is a flavoprotein that catalyzes the oxidation of hypoxanthine to xanthine and generates superoxide and uric acid. Indeed, the results obtained indicate that the methanolic extract presented the best inhibitory activity of 4.85 ± 0.80 *μ*g/mL. Similarly, the methanolic extract has a significantly (*p* < 0.05) higher activity than the ethanolic (7.80 ± 1.5 *μ*g/mL) and aqueous (7.83 ± 1.21 *μ*g/mL) extracts. On the other hand, there is no difference in inhibitory activity between the aqueous and ethanolic extracts (Table 4).

#### 3.4.2. *In Vivo* Studies

*(1) (1) Lipid Peroxidation*. The effect of aqueous and alcoholic extracts of *E. guttatum* on lipid peroxidation in hepatic, renal, and pancreatic tissues in mice rendered diabetic by HFD-STZ is summarized in Table 5. Indeed, we found an elevation of MDA in the liver, kidneys, and pancreas of untreated diabetic mice (0.961 ± 0.07 nM/g liver; 0.813 ± 0.08 nM/g liver; 0.861 ± 0.09 nM/g liver), respectively, compared to the nondiabetic control group (0.36 ± 0.03 nM/g liver; 0.24 ± 0.02 nM/g liver; 0.402 ± 0.02 nM/g liver). Likewise, *E. guttatum* extracts reduced MDA levels compared to the control (nondiabetic) group. The diabetic group treated with the methanolic extract showed a better reduction in MDA. In addition, in the liver and kidney, there was a significant difference between the group treated with methanol extract (0.188 ± 0.03 nM/g liver; 0.195 ± 0.03 nM/g kidney) and the group treated with metformin (0.55 ± 0.06 nM/g liver; 0.42 ± 0.03 nM/g kidney) (*p* < 0.05). The control (nondiabetic) group was significantly inferior to the metformin-treated group (*p* < 0.05) in the liver and kidney.

*(2) (2) Superoxide Dismutase (SOD), Catalase (CAT), and Glutathione (GSH) Activity*. The extracts of *E. guttatum* enhanced the production of enzymatic and nonenzymatic antioxidants (SOD, CAT, and GSH, respectively). The results are presented in Tables 6, 7, and 8. For three tests, we found that the groups of mice treated with the methanolic extract showed better secretion of enzymatic and nonenzymatic antioxidants. Also, we noticed a better increase in SOD secretion in the groups of diabetic mice treated with the methanolic extract and metformin compared to the normal control group (*p* < 0.05). Similarly, the enzymatic activity of SOD on hepatic, renal, and pancreatic tissues was significantly reduced in untreated diabetic mice compared to that measured in normal controls (*p* < 0.05). On the other hand, diabetic mice treated with ethanolic and aqueous extracts caused an increase in SOD levels in the kidneys and pancreas, indeed, for ethanolic extract (799 ± 8.38 units/g of the kidney; 616.3 ± 1.44 units/g of the pancreas) and for the aqueous extract (768.2 ± 3.1 units/g of the kidney; 565.9 ± 1.92 units/g of pancreas).

In addition, in the three organs (liver, kidney, and pancreas), the level of CAT was increased in the groups of diabetic mice treated with the aqueous, ethanolic, and methanolic extracts. Similarly, the treatment of diabetic mice with extracts of *E. guttatum* caused an increase in the level of CAT compared to the groups of mice treated with metformin (*p* < 0.05). Thus, there was no significant difference between the normal (nondiabetic) control group and the metformin-treated group (*p* < 0.05). Similarly, there was no significant difference between the groups of diabetic mice treated with the methanolic extract (0.46 ± 0.23 U/mg of protein) and the ethanolic extract (0.46 ± 0.11 U/mg protein) (*p* > 0.05) in the liver.

Similarly, in the liver and kidney, the GSH level was significantly increased in the groups of diabetic mice treated with the three extracts. In the pancreas, the level of GSH in the groups of mice treated with the aqueous extract (77.0 ± 0.05 *μ*mol/mg of protein) is significantly higher than that of the groups treated with the methanolic extract (67.4 ± 0.0305 *μ*mol/mg of protein) and ethanol (62.2 ± 0.0305 *μ*mol/mg of protein) as well as the group treated with metformin (63.3 ± 0.0505 *μ*mol/min/mg protein) and the untreated group (65.6 ± 0.0305 *μ*mol/mg protein) (*p* < 0.05).

## 4. Discussion

Here, we reported mineral and phenolic composition of *E. guttatum* root extracts as well as their antioxidant and antidiabetic activities. Mineral composition analysis showed that *E. guttatum* roots are rich in Ca^2+^ (28300.88 mg/kg), K^+^ (8861.61 mg/kg), Mg (2421.03 mg/kg), and P (1421.74 mg/kg) with minor content of Se (0.01 mg/kg), Cd (0.12 mg/kg), and Mo (0.5 mg/kg). Indeed, these minerals are essential for restoring solid tissues in bones and teeth, as well as regenerating blood cells and maintaining osmotic balance [29]. K^+^ maintains the ionic balance and excitability of tissues, Ca^2+^ is an anticoagulant and necessary for the functioning of the myocardium, and Fe^2+^ plays an important role. It is involved in the binding of oxygen to hemoglobin and acts as a catalyst for several enzymes such as cytochrome oxidase. Magnesium protects muscle cells from degeneration, growth retardation, cardiomyopathy, immunological dysfunction, impaired spermatogenesis, and bleeding disorders [28, 29]. Chromium is one of the indispensable metals in the human body and animals. It is involved in the regulation of glucose tolerance [30–32]. For Pd, it has been reported that the permissible and accepted daily intake by WHO is 10 mg/kg. Thus, the roots of *E. guttatum* have a concentration lower than the allowed concentration [33]. The abundance of Ca^2+^, K^+^, and Mg is similar to other previous studies in plants [32–35].

The phytochemical assay reveals that methanolic extract is the richest in polyphenols, tannins, and flavonoids, with a content of 389.20 ± 1.55 mg of EAG/g of extract, 289.70 ± 3.57 mg EC/g of the extract, and 432.5 ± 3.21 mg of RE/g of the extract. However, these results can be explained by the difference in polarity of the solvents used to extract the phenolic molecules. These values are higher than those obtained by Hamza et al. [13] who worked on the same species from a different region. It was reported that *E. guttatum* hydromethanolic extract contains 124 ± 6 mg GAE/g extract of total polyphenols, 52 ± 2.3 d ER/g extract of flavonoids, and 20 ± 0.5 mg EC/g extract of tannins. Additionally, phenols and phenolic compounds, including flavonoids, have been reported to be widely present in plant sources and to promote antioxidant potency [36].

Chemical analysis by ESI-HRMS identified certain bioactive compounds such as shikimic acid, rottlerin, rugulosin, procyanidin C1, and vanillic acid. In addition, several scientific studies demonstrate the antidiabetic power of natural resources [37]. For example, shikimic acid is an intermediate metabolite synthesized from tryptophan that induces specific anabolic effects on bone, decreases oxidative stress, and reduces the expression of antioxidant genes in the diabetic retina of rats. Also, it decreases the formation of advanced glycation end products derived from glucose [38]. Gallic acid has antihyperglycemic, antilipid peroxidative, and antioxidant effects. It regulates mitochondrial function *via* the activation of the alpha receptor which are activated by the coactivator1 alpha peroxisome proliferators (PGC1 alpha) and increases the translocation of GLUT4 and therefore the activity of glucose absorption in an independent manner. Gallic acid prevents the damage caused by oxidative stress in the diabetic state [39]. Catechin maintains a low level of hemoglobin A (1c) in type 2 diabetics, improves the level of glucokinase, glucose-6 phosphatase, glycogen synthase, and glycogen phosphorylase, decreases the level of cholesterol and triglycerides, and induces the restoration of the structure of the artery wall and cerebral blood flow [40]. Moreover, other studies have also reported the antidiabetic effect of vanillic acid [39, 40].

The results obtained about antiradical activity *E. guttatum* root extracts show that methanolic extract has the strongest DPPH radical inhibitory activity, with an IC_50_ of 3 ± 1.45 *μ*g/mL. This same extract has the most powerful iron-reducing power (540.2 ± 1.40 mg EAA/gE), which was observed with the FRAP test. This extract also has the ability to stabilize the highest ABTS cationic radical (228.68 ± 2.93 mg ET/gE). Also, the same result was observed by the xanthine oxidase test. On the other hand, the study of the antioxidant activity with H_2_O_2_ trapping test indicated that aqueous extract has the strongest inhibiting activity of the H_2_O_2_ radical with an IC_50_ of 4.65 ± 0.7 *μ*g/mL. These different tests revealed that *E. guttatum* extracts have significant antioxidant effects, with minor differences between aqueous and alcoholic extracts. Moreover, these differences between these three extracts could be explained by the number of phenolic compounds such as polyphenols and flavonoids as well as the type of active ingredient present in each extract [41–44]. Moreover, by comparing our results with other results obtained for the same species, we found that the antioxidant activities of the root extracts of *E. guttatum* are more potent than those obtained by Hamza et al. [13].

In order to confirm the antioxidant effect *in vitro*, we studied the antioxidant effect *in vivo* on hepatic, renal, and pancreatic tissue in mice rendered diabetic by HFD-STZ. We found that the methanolic extract was the best extract that reduced MDA levels and therefore can inhibit lipid peroxidation, with a statistically insignificant difference compared to metformin. Similarly, we noticed an increase in the production of the MDA marker in untreated diabetic mice.

For the determination of enzymatic and nonenzymatic antioxidants (SOD, CAT, and GSH, respectively), we noticed an increase in antioxidant enzymes in diabetic mice treated with *E. guttatum* extracts with better secretion obtained by the methanolic extract. Indeed, lipid peroxidation is promoted by the generation of free radicals, which in turn produce end products such as malondialdehyde [45]. During oxidative stress, the level of peroxidation end products such as aldehydes increases, and the production of this biomarker is used in the measurement of oxidative stress [44, 45]. Thus, several modes of action of lipid peroxidation have been reported in the literature [46, 47]. These results obtained are similar to previous studies demonstrating the lipid peroxidation inhibitory effect of different plant extracts [48, 49]. Likewise, it has been reported that this activity can be attributed to the phenol content, which correlates with their chemical structures.

Superoxide dismutase (SOD) is one of the important enzymes of the antioxidant defense system. It removes by converting superoxide anions into hydrogen peroxide, thereby reducing toxicity caused by reactive oxygen species [50, 51]. Catalase is another antioxidant enzyme that catalyzes hydrogen peroxide into water and oxygen, protecting tissues from the deleterious effects of hydroxyl radicals. Glutathione (GSH) is a nonenzymatic tripeptide that hydrolyzes free radicals directly and protects proteins against free radicals [52, 53]. Treatment of diabetic mice with *E. guttatum* extracts showed an increase in the content of these enzymes in animal tissues. The extracts also increased the content of GSH protein. This indicates that these extracts may improve enzymatic activities by increasing the content of CAT, SOD, and GSH. In addition, it has been reported in several studies that medicinal plants can restore and activate the content of antioxidant enzymes in streptozotocin-induced diabetic animals [32, 54–56].

## 5. Conclusion

The phytochemical assay and the evaluation of the mineral composition of *E. guttatum* aqueous and alcoholic extracts showed their richness in phenolic compounds (polyphenols, flavonoids, and tannins) and minerals. Similarly, the study of *in vitro* antioxidant activity using five methods (DPPH, ABTS, FRAP, H_2_O_2_, and xanthine oxidase) and *in vivo* (by the dosage of MDA, SOD, CAT, and GSH) showed that plant extracts, particularly the methanolic extract, exhibit remarkable antioxidant activity. Moreover, the results indicate a positive correlation between the antioxidant activity and the content of phenolic compounds. Moreover, the reducing and antiradical activities are well correlated; this means that the substances present in the plant extracts studied have the property of reducing oxidants and trapping free radicals. Therefore, the obtained data provides a basis for further toxicological and pharmacological studies.

## Figures and Tables

**Table 1:** Mineral composition of *Erodium guttatum* roots.

Mineral composition (mg/kg)	*E. guttatum*
B	19.54
Ca	28300.88
Cu	15.64
Fe	170.54
K	8861.61
Mg	2421.03
Mn	18.15
Na	228.26
P	1421.74
Zn	334.74
As	4.12
Cd	0.12
Co	3.45
Cr	5.12
Mo	0.5
Ni	7.14
Pb	5.87
Se	0.01
V	7.14

**Table 2:** Total phenolic, flavonoid, and condensed tannin contents of *E. guttatum*.

		Phenolic content^(1)^	Flavonoids content^(2)^	Tannin content^(3)^
*Erodium guttatum*	Aqueous extract	248.17 ± 1.81	315.5 ± 4.50	114.95 ± 2.60
	Methanol extract	389.20 ± 1.55	432.5 ± 3.21	289.70 ± 3.57
	Ethanol extract	348.1 ± 2.3	417.5 ± 1.42	243.3 ± 2.21

The results are expressed as ^(1)^ mg of gallic acid equivalent, ^(2)^ mg of rutin equivalent, and ^(3)^ mg of catechin equivalent.

**Table 3:** Data from aqueous, ethanolic, and methanolic extracts of *E. guttatum* root by ESI-HRMS experiments.

*m*/*z*	Name	Formula	RDB equiv
170	Gallic acid	C_6_H_6_O_5_	5.5
290	Catechin	C_15_H_14_O_6_	9.5
516	Rottlerin	C_30_H_28_O_8_	17.5
866	Procyanidine C1	C_45_H_38_O_18_	27.5
120	Purine	C_5_H_4_N_4_	6.5
174	Shikimic acid	C_7_H_10_O_5_	3.5
542	Rugulosin	C_30_H_22_O_10_	19.5
168	Vannilic acid	C_8_H_8_O_4_	5.5
342	Sucrose	C_12_H_22_O_11_	2.5
594	Tilirozide	C_30_H_26_O_13_	17.5

**Table 4:** Antioxidant activity by DPPH, FRAP, ABTS, H_2_O_2_, and xanthine oxidase (XO) methods of *Erodium guttatum*; average of three replicates.

		DPPH test^(1)^	ABTS test^(2)^	FRAP test^(3)^	H_2_O_2_ test^(1)^	Xanthine oxidase (XO)^(1)^
*Erodium guttatum*	Aqueous extract	7 ± 3.78^∗∗^	218.52 ± 1.34^∗∗^	338.7 ± 0.56^∗^	4.65 ± 0.7^∗∗∗∗^	7.83 ± 1.21^∗∗∗∗^
	Methanol extract	3 ± 1.45^∗∗∗^	228.68 ± 2.93^∗∗∗∗^	540.2 ± 1.40^ns^	7.43 ± 1.58^∗∗^	4.85 ± 0.80^∗∗^
	Ethanol extract	4 ± 1.24^∗∗∗^	189.13 ± 1.41^∗∗∗∗^	220.72 ± 1.05^ns^	5.21 ± 0.68^∗∗∗∗^	7.80 ± 1.5^∗∗∗∗^
BHT		3.28 ± 0.79	—	—	—	—
Ascorbic acid		—	—	—	5.98 ± 0.47	—
Allopurinol		—	—	—	—	0.78 ± 0.01

The results are expressed as ^(1)^ IC_50_ (*μ*g/mL), ^(2)^ mg of Trolox equivalent, and ^(3)^ mg of ascorbic acid equivalent per gram of extracts. Data are the mean ± standard deviation. Values are mean ± SEM. ^∗∗∗∗^Highly significant statistical difference (*p* < 0.0001), ^∗∗∗^ (*p* < 0,001), ^∗∗^ (*p* < 0.01), ^∗^ (*p* < 0.5); ns: statistically insignificant difference.

**Table 5:** Malondialdehyde (MDA) levels in the liver, kidney, and pancreas in diabetic mice treated with extracts of *E. guttatum* (200 mg/kg) and metformin (300 mg/kg) (nM/mg tissue).

	EGA	EGM	EGE	DT	ND	DTM
Liver	0.259 ± 0.03^a,c^	0.188 ± 0.03^a^	0.216 ± 0.03^a^	0.961 ± 0.07^b^	0.36 ± 0.03^c^	0.55 ± 0.06^d^
Kidney	0.325 ± 0.05^e,h,i^	0.195 ± 0.03^f^	0.251 ± 0.04^e,f^	0.813 ± 0.08^g^	0.24 ± 0.02^f,h^	0.42 ± 0.03^i^
Pancreas	0.211 ± 0.01^j^	0.180 ± 0.02^j^	0.209 ± 0.02^j^	0.861 ± 0.09^k^	0.402 ± 0.02^j,l^	0.410 ± 0.04^l^

Data represent the mean ± standard deviation of six independent experiments. Values in the same line with different superscript letters indicate significant differences (*p* value < 0.0001). Abbreviations: EGA: aqueous extract of *E. guttatum*; EGM: methanolic extract of *E. guttatum*; EGE: ethanolic extract of *E. guttatum*. DT: diabetic group; ND: nondiabetic group; DTM: metformin-treated diabetic group.

**Table 6:** Level of the antioxidant superoxide dismutase enzyme (SOD) in the liver, kidney, and pancreas in diabetic mice treated with extracts of *E. guttatum* (200 mg/kg) and metformin (300 mg/kg) (unit/g tissue).

	EGA	EGM	EGE	DT	ND	DTM
Liver	638.7 ± 6.21^a^	793.1 ± 0.85^b^	702.5 ± 0.95^c^	532.3 ± 2.86^d^	732.2 ± 11.7^e^	904.3 ± 7.8^f^
Kidney	768.2 ± 3.1^g^	811.4 ± 2.17^h,i^	799 ± 8.38^h^	420.5 ± 10.2^j^	830.0 ± 3.06^i,k^	832.1 ± 9.2^k^
Pancreas	565.9 ± 1.92^l^	632.4 ± 3.75^m^	616.3 ± 1.44^m^	480.2 ± 22.5^n^	540.6 ± 12.5^l^	724.2 ± 6.3^o^

Data represent the mean ± standard deviation of six independent experiments. Values in the same line with different superscript letters indicate significant differences (*p* value < 0.0001). Abbreviations: EGA: aqueous extract of *E. guttatum*; EGM: methanolic extract of *E. guttatum*; EGE: ethanolic extract of *E. guttatum*. DT: diabetic group; ND: nondiabetic group; DTM: metformin-treated diabetic group.

**Table 7:** Catalase (CAT) levels in the liver, kidney, and pancreas in diabetic mice treated with extracts of *E. guttatum* (200 mg/kg) and metformin (300 mg/kg) (U/mg protein).

	EGA	EGM	EGE	DT	ND	DTM
Liver	0.43 ± 0.05	0.46 ± 0.23	0.46 ± 0.11	0.18 ± 0.06	0.48 ± 0.04	0.47 ± 0.04
Kidney	0.46 ± 0.02	0.54 ± 0.02	0.49 ± 0.02	0.14 ± 0.03	0.56 ± 0.02	0.5 ± 0.06
Pancreas	0.51 ± 0.02	0.59 ± 0.01	0.54 ± 0.03	0.17 ± 0.08	0.43 ± 0.04	0.42 ± 0.02

Values are mean ± SD. Values are mean ± SEM. ns: statistically insignificant difference. EGA: aqueous extract of *E. guttatum*; EGM: methanolic extract of *E. guttatum*; EGE: ethanolic extract of *E. guttatum*; DT: diabetic group; ND: nondiabetic group; DTM: diabetic group treated with metformin.

**Table 8:** Glutathione (GSH) levels in the liver, kidney, and pancreas in diabetic mice treated with extracts of *E. guttatum* (200 mg/kg) and metformin (300 mg/kg) (*μ*mol/min/mg of proteins).

	EGA	EGM	EGE	DT	ND	DTM
Liver	79.1 ± 0.06^a^	81.5 ± 0.02^b^	71.3 ± 0.07^c^	41.7 ± 0.03^d^	89.0 ± 0.03^e^	85.4 ± 0.05^f^
Kidney	70.3 ± 0.05^g^	79.6 ± 0.05^h^	77.0 ± 0.04^i^	35.2 ± 0.05^j^	84.3 ± 0.05^k^	87.0 ± 0.02^l^
Pancreas	77.0 ± 0.05^m^	67.4 ± 0.03^n^	62.2 ± 0.03^o^	33.2 ± 0.09^p^	65.6 ± 0.03^q^	63.3 ± 0.05^r^

Data represent the mean ± standard deviation of six dependent experiments. Values in the same line with different superscript letters indicate significant differences (*p* value < 0.0001). Abbreviations: EGA: aqueous extract of *E. guttatum*; EGM: methanolic extract of *E. guttatum*; EGE: ethanolic extract of *E. guttatum*. DT: diabetic group; ND: nondiabetic group; DTM: metformin-treated diabetic group.

## Data Availability

The data used to support the findings of this study are included within the article.
